# Improving LGBTQ+ Inclusive Care in Long-Term Care: Staff perspectives, Barriers, and Recommendations

**DOI:** 10.1093/geroni/igaf122.2773

**Published:** 2025-12-31

**Authors:** Andrew Alberth

**Affiliations:** University of Massachusetts Boston, Boston, Massachusetts, United States

## Abstract

Lesbian, gay, bisexual, and transgender (LGBTQ+) older adults experience lifetime discrimination in healthcare settings, leading to worse health outcomes and lower trust in healthcare providers. Discrimination-related health outcomes may increase reliance on long term care (LTC) among LGBTQ+ older adults. To ensure LGBTQ+ residents get discrimination-free care, LGBTQ+ older adults often hide their sexual and gender identities. As such, little is known about how LTC staff identify and provide care to LGBTQ+ residents. This study aimed to identify LTC staff’s perspectives on LGBTQ+ nursing home and assisted living facility residents’ care and ways to improve that care. Semi-structured, in person, virtual, and phone-based interviews from 34 nursing home and assisted living facility direct care staff and administrators were recorded and transcribed. Thematic analysis identified common barriers and facilitators staff face when caring for LGBTQ+ residents and recommendations for improving LTC for this population. Barriers facing LTC staff include a lack of training on LGBTQ+-specific care needs, identifying LGBTQ+ residents, and navigating interactions with LGBTQ+ residents. Facilitators to staff caring for LGBTQ+ residents include a desire to provide high quality care to all residents and having LGBTQ+ staff and residents present in facilities. LTC staff recommended LGBTQ+ training for staff and residents, adding LGBTQ+ symbols in promotional materials and décor throughout the facility, and building opportunities for socialization and connection. This study explored LTC staff’s perspectives on care provided to LGBTQ+ residents and recommendations to improve their care. Additional research is necessary to understand LGBTQ+ residents’ perspectives in LTC.

